# Systemic Lupus Erythematous: Gene Polymorphisms, Epigenetics, Environmental, Hormonal and Nutritional Factors in the Consideration of Personalized Therapy

**DOI:** 10.26502/aimr.0188

**Published:** 2024-12-24

**Authors:** Nejma Wais, Devendra K. Agrawal

**Affiliations:** 1 Department of Translational Research, College of Osteopathic Medicine of the Pacific, Western University of Health Sciences, Pomona, California 91766 USA

**Keywords:** Autoimmune disease, Environmental factors, Epigenetics, Gene polymorphism, Nutritional factors, Pathophysiology, Systemic Lupus Erythematosus

## Abstract

Systemic Lupus Erythematosus (SLE) is a chronic illness that can affect many tissues through the production of autoantibodies. A definite etiology has not been conclusively established, but current research points to the influences which include genetic, hormonal and environmental factors. SLE is difficult to treat due to its multifactorial pathogenesis and heterogeneity in clinical manifestations. Current treatment mainly includes anti-malarial medications, glucocorticoids, and biologics, but many patients still struggle in achieving remission. Additionally, there is no definite cure for SLE as of now, which further emphasizes the need for personalized treatment approaches. We analyzed genetic polymorphisms, DNA methylation, and other environmental, hormonal and nutritional factors in the development of SLE. We considered how such factors affect the processes of the disease pathogenesis and may provide insight on targets for potential personalized therapy. In this article, we criticaly reviewed the literature for compelling evidence connecting SLE and specific genes and epigenetic changes. We also explored environmental triggers such as UV exposure, and hormonal influences on their connection to SLE, working toward understanding the disease’s complex nature. A critical evaluation is presented on the use of already accredited biologics in SLE that are beneficial to patients, including anifrolumab and belimumab. The reports on many factors that may influence SLE pathophysiology, along with success with recent biologics/targeted therapies, suggest that precision medicine, tailored to individual genetic and environmental profiles, may hold promise for enhancing remission rates and quality of life for SLE patients. The findings contribute to the field by addressing the need for an integrative approach to SLE treatment and offer more evidence for the potential critical benefit of personalized management strategies that may provide long-term solutions in this challenging and complex disease.

## Introduction

**Figure 1:**
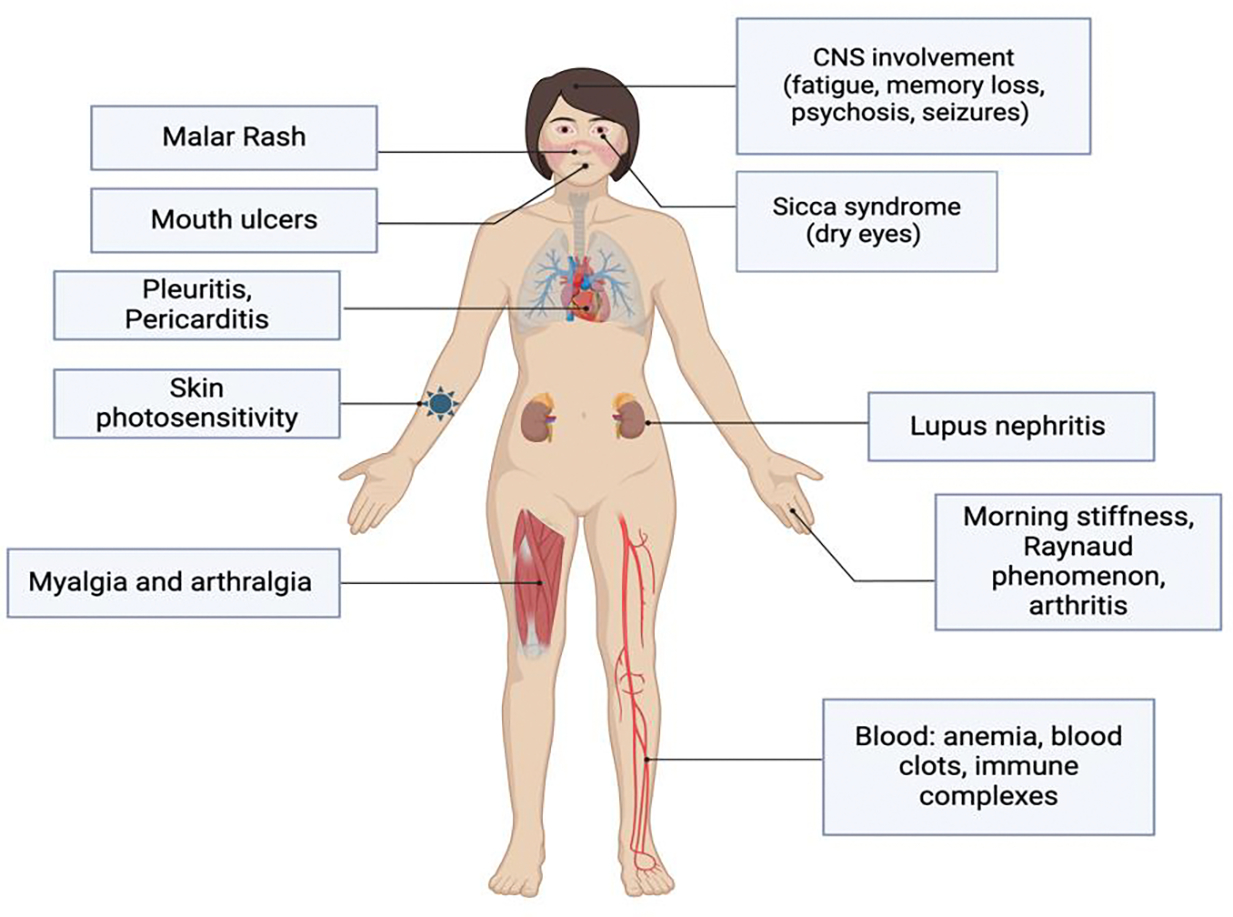
Manifestations of Systemic Lupus Erythematosus. Created with BioRender.com.

Systemic Lupus Erythematosus (SLE) is a chronic autoimmune disorder, in which the immune system attacks its own body tissues, leading to widespread inflammation and permanent damage in some cases (Figure 1). There are four main types of lupus, with SLE being the most common type, affecting more than 200,000 people in the United States. Women of reproductive age are largely affected by SLE, with 9 out of 10 people with lupus being women [1]. Additionally, the disease disproportionately affects certain ethnic groups, with higher prevalence rates observed among African American, Hispanic, and Asian populations compared to Caucasians [2]. The exact etiology underlying SLE is unknown, but likely involves a combination of genetic, hormonal, and environmental influences [3]. Approximately 10–12% of SLE patients have first-degree relatives with autoimmune disorders, highlighting a strong heritable component [4], with some studies showing heritability as high as 43.9% [5]. The pathophysiology underlying SLE is complex, and the disease presents differently in patients making diagnosis and treatment challenging. Thus, defining effective management strategies for SLE remains difficult, highlighting the importance of tailored treatment approaches that accommodate unique disease manifestations of each patient [6].

Due to its autoimmune nature, the primary treatment strategy for SLE involves the use of anti-inflammatory and immunosuppressive medications. The classes of medications used to treat SLE include antimalarials such as chloroquine and hydroxychloroquine, glucocorticoids, non-corticosteroid immunosuppressants such as cyclophosphamide, mycophenolate, azathioprine, and methotrexate, and calcineurin inhibitors such as voclosporin. Non-steroidal anti-inflammatories are also commonly used to reduce inflammation and pain associated with SLE flares. More recently, biologic therapies have been utilized to manage SLE, especially in patients who do not respond to traditional therapies. Biologics that have been approved for treatment of SLE are anifrolumab and belimumab, with rituximab being used off-label [7]. Additionally, anticoagulants can be utilized in patients with antiphospholipid antibodies [8]. Most patients with SLE take medications throughout their lives, however many of these medications, such as chronic glucocorticoid use, cause complications. It is also important to note that there is still no definitive treatment for SLE. A personalized risk-benefit approach should be utilized for SLE patients to manage disease symptoms while limiting the side effects from medication use, which further adds to the complex management of the disease.

In many patients, a combination of the medications above are utilized. However, despite advancements in treatment, achieving and sustaining remission remains a challenge in SLE. Remission is when signs and symptoms of lupus disappear for an extended period of time and is based on the Definition of Remission in SLE (DORIS) criteria. However, in clinical practice, remission is rarely achieved; therefore, the Lupus Low Disease Activity State (LLDAS) serves as a more feasible treatment goal for a larger number of patients. Recent studies show that 42.4% to 88% of patients with SLE can achieve and maintain remission for a year, while 21.1% to 70% stay in remission for at least five years. Patients more likely to reach remission tend to be older at diagnosis, have milder initial disease activity, and have no major organ involvement. When remission is achieved, especially for longer periods, it generally leads to less disease-related damage and a better quality of life for people with SLE [9].

There have been robust efforts to strive toward increased remission in SLE, including research into biomarkers for nonresponse or response to current treatments, genetic polymorphisms and epigenetic contributions. We criticaly reviewed and summarized the current efforts in identifying genetic polymorphisms, epigenetics, environmental factors, hormonal factors, and nutritional factors that contribute to the pathophysiology of SLE. We also discussed the role of biologics and targeted therapies that could target these factors to increase remission, as well as discussing research on the gut microbiome which may lead to improved outcomes in SLE.

## Key Factors Impacting the Pathophysiology of SLE

### A. Cellular and Immuno-molecular Factors

#### (A.1) Genetic Polymorphisms in the Human Leukocyte Antigen (HLA) Region:

HLA are genes in major histocompatibility complexes. They help encode proteins that are essential for distinguishing self from non-self and are important in immune defense. However, they can contribute to certain autoimmune diseases as well, such as SLE. There are three main types of HLA: Class I, Class II, and Class III. Class I includes HLA-A, B, and C, while class II includes HLA-DR, -DQ, and –DP. Class III encodes for proteins important in inflammation such as the complement system (10). SLE has been strongly associated with class II HLA, specifically HLA-DR2, HLA-DR3, HLA-DRB1, and HLA-DQA1, with predominance of certain alleles varying by ancestral descent [11]. It has been thought that the reason these HLA alleles are associated with SLE is due to the presentation of self-antigens that lead to adaptative immune system-mediated damage. Recent research shows that allele DRB1*03:01 encodes an epitope that can trigger a cascade of SLE events independent of antigen presentation and has characterized this allele as the most significant HLA allele associated with SLE among many different populations [12]. These findings offer a direction of exploration for new therapeutic treatments in SLE.

#### (A.2) The Complement System:

**Figure 2:**
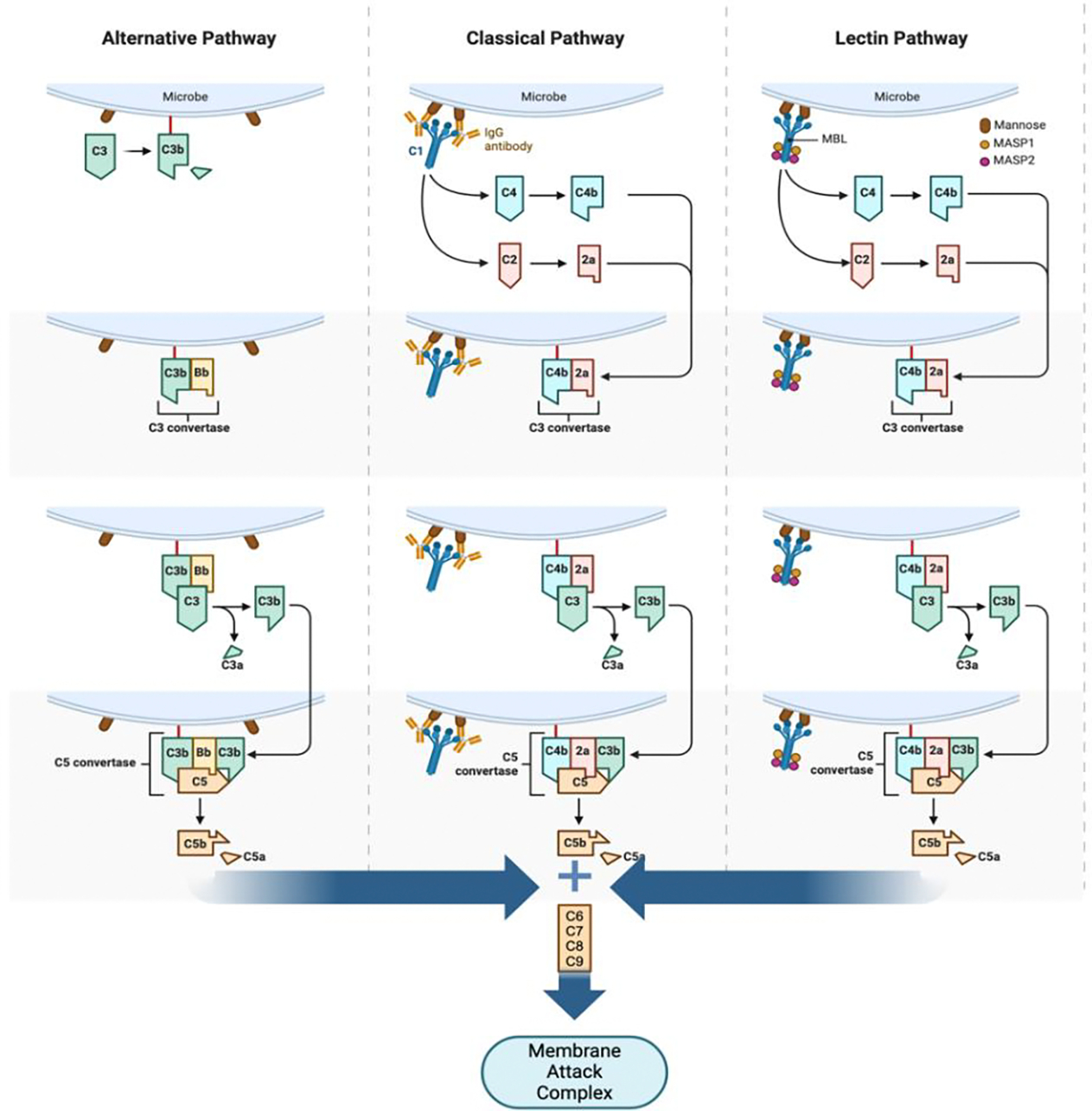
The complement pathway. Binding of complement proteins to microbial cell surface or antibody. Formation of C3 convertase. Cleavage of C3 by C3 convertase. Formation of C5 convertase. Formation of Membrane Attack Complex (MAC). Created with BioRender.com.

The complement system is a part of the immune system and consists of proteins that enhance the body’s ability to recognize, target, and destroy pathogens, aiding in inflammation and immune response. Complement proteins can be activated by three pathways: the classical pathway, the alternative pathway, and the lectin pathway (Figure 2). Activation of the complement system has been implicated in SLE, resulting in tissue damage in different organs. Low levels of the complement proteins C3 and C4 have been associated with SLE, and for years these proteins have been utilized to gauge SLE disease activity [13]. Low serum complement levels have also been used in certain classification criteria of SLE, signifying the importance of the complement system in SLE pathophysiology. However, low complement levels have been implicated in other autoimmune diseases as well, complicating the specificity and sensitivity of these markers. In SLE, the classical pathway is activated, which presents with decreased serum levels of C1q (90–93%), C1r/s (50–57%), C4 (75%), and C2 (10%) (14). C3 has also been shown to be low in SLE, however the levels tend to remain normal due to C4 binding protein inhibiting the classical pathway. It is important to note that low complement levels have not been associated with disease flares [14]. Furthermore, recent studies have shown that variants of the C1q gene have also been associated with SLE, particularly in cases with lupus nephritis, however additional studies should be done to advance research in this area [15].

#### (A.3) Tumor Necrosis Factor Alpha (TNF-α):

**Figure 3:**
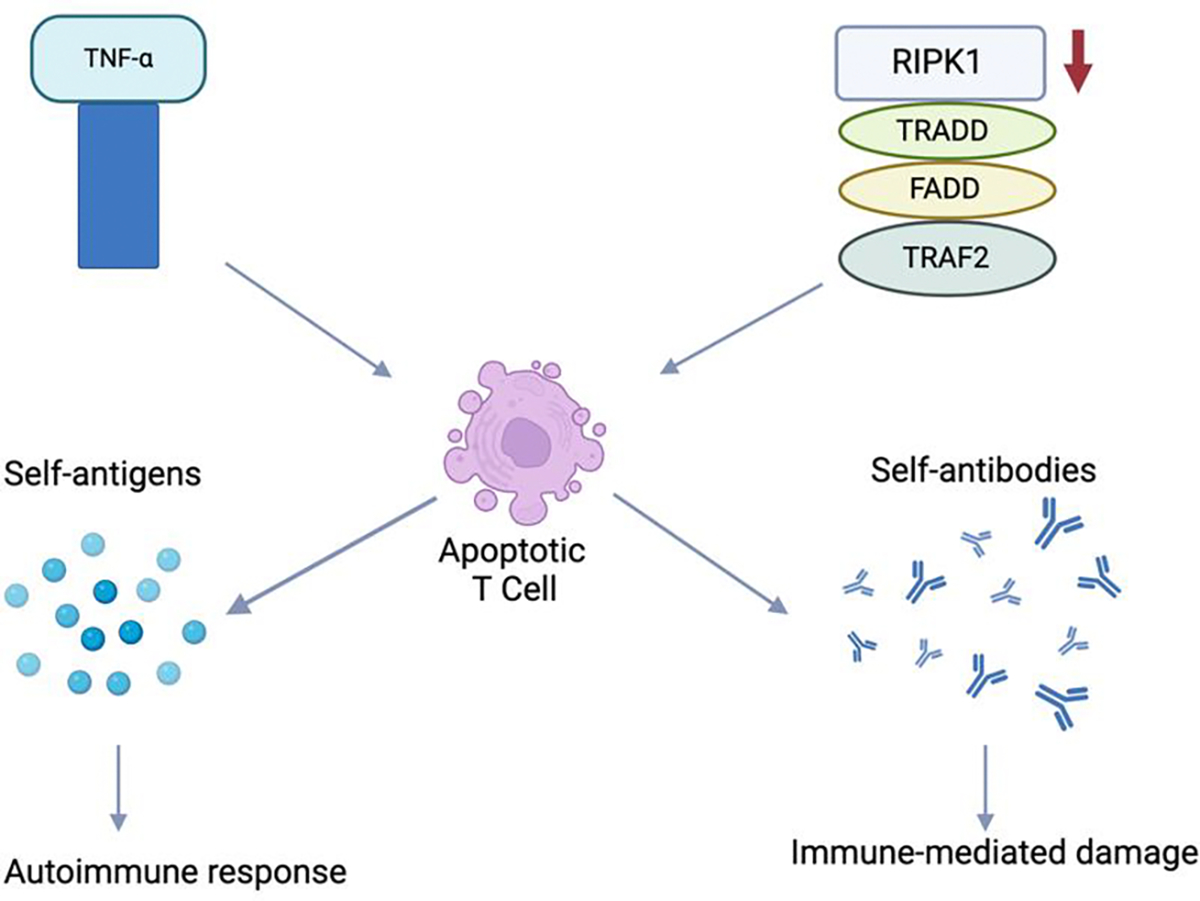
Role of TNF-α in systemic lupus erythematosus. RIPK1, receptor-interacting serine/threonine-protein kinase 1; TRAF2, TNFR-associated factor 2; TRADD, TNFR-associated death domain protein; FADD, Fas-associated protein with death domain. Created with BioRender.com.

TNF-α is a protein that is expressed on activated macrophages, T-lymphocytes, and killer cells. It regulates inflammatory responses by triggering molecules such as cytokines (include figure here, example included in the bottom). The function of TNF-α is complex; it is known to combat certain disease processes and was initially recognized to be involved in the necrosis of tumors. However, TNF-α is also implicated in the pathogenesis of autoimmune diseases such as rheumatoid arthritis, psoriatic arthritis, and irritable bowel disease for which TNF-α inhibitors can be utilized for treatment [16]. The role of TNF-α in SLE is not yet fully understood; some studies have concluded that higher TNF-α levels contribute to the development of SLE [17] while others have suggested that TNF-α may be a protective factor in SLE [18]. Recent research [19] has also elaborated on the conflicting evidence on the role of TNF-α in SLE, with studies showing elevated levels of TNF-α implicated in SLE [20], relation between TNF- α promoter polymorphism and SLE susceptibility [21], and increased TNF-α levels found to be associated with disease severity [22] all supporting the role of increased TNF-α levels in SLE pathophysiology. One study found that SLE patients exhibit significantly reduced levels of TNF-α adapter proteins, including TRADD, FADD, TRAF-2, and RIPK-1, in peripheral blood mononuclear cells, which correlate inversely with disease activity. These low levels are associated with increased lymphocyte apoptosis and high autoantibody production, leading to immune-mediated damage in SLE patients (20; Figure 3). However, some research has suggested decreased expression of TNF-α are correlated with disease activity in SLE [23]. This makes the use of medications targeting TNF-α controversial in SLE, and further research should be done in this area to assess if this would be a successful therapeutic target for SLE treatment.

#### (A.4) Nuclear Factor Kappa B (NF-κB) Pathway:

NF-κB is a part of a family of transcription factors, and there are to different pathways for NF-κB, the canonical and the non-canonical pathways. The canonical pathway responds to external stimuli involved in inflammation and the immune response, whereas the non-canonical pathway is activated through TNF superfamily receptors [24]. It has been postulated that patients with SLE rely on the non-canonical NF-κB pathway for the pathological survival and differentiation of autoantibody-producing B cells [25]. A recent study evaluated the activation of the NF-κB pathway in microparticles isolated from patients with SLE vs healthy controls, since elevated levels of circulation microparticles (MPs) have been reported in patients with SLE. The findings showed that the MP-induced activation of monocytes perpetrated the inflammatory state in patients with SLE, at least in part mediated by the NF-κB pathway [26]. Furthermore, NF-κB has been implicated as a player in lupus nephritis, the leading cause of morbidity and mortality from SLE [27]. This study showed that variants in numerous genes involved in the TLR/NF-κB signaling axis were associated with lupus nephritis, suggesting that inhibition of these variants could provide therapeutic treatment for lupus nephritis patients who have variants in NF-κB regulatory genes. The use of monoclonal antibodies that inhibit NF-κB effector molecules such as IFNs, IL-6, IL-17, IL-23, and IP-10, which are implicated in lupus nephritis, is another area of possible precision treatment [28], which has led to the study and use of Anifrolumab, a FNAR1 antagonist, in SLE treatment. We will discuss its use in Biologics and Targeted Therapies section below.

#### (A.5) Interferon (IFN) Pathway Genes:

It is well known that SLE is characterized by the activation of the IFN system. IFNs are immunomodulatory substances produced in the response to pathogens, such as in viral and bacterial infections [29]. This system is monumental in the human defense of infections; however, in patients with SLE, the ongoing production of IFNs participate in sustaining the autoimmune process. There are three subtypes of IFNs: alpha, beta, and gamma. IFN-alpha and IFN-beta belong to the Type I IFN group. IFN-gamma belongs to the Type II IFN group [30]. Type I IFNs have been implicated in the pathogenesis of SLE. The main Type I IFN producing cell is the plasmacytoid dendritic cell (pDC), however several cell types have been shown to contribute to the IFN signature in SLE patients [31]. In recent years, there has been research that shows IFN-related genetic variants are an important part of SLE pathogenesis, such as IRF5, IRF7, IRF8, STAT4, PTPN22, OPN/SPP1, IFIH1, and TYK2 [32]. Another study showed that Type I IFN gene expression is associated with the dermatologic, musculoskeletal, renal, vascular, central nervous system and hematological manifestations of SLE [33]. These studies, among numerous others, show the importance of IFNs in the pathogenesis of SLE, which has led to research on biologics that target IFN, with the eventual development and use of Anifrolumab, a Type I IFN blocking antibody, in SLE.

#### (A.6) Apoptosis and Clearance Genes:

Apoptosis is a form of programmed cell death for cells that are no longer required or pose a threat (34). In SLE patients, there has been observation of accelerated apoptosis of circulating cells with lupus autoantigens such as dsDNA being exposed on surface blebs. It has been thought that this accelerated apoptosis may be due to alterations in genes such as Fas and Bcl-2, which are proteins that play roles in cell death. Fas initiates a signaling cascade that can lead to apoptosis, and increased expression of the Fas antigen may intensify the exposure of hidden antigens in SLE, while Bcl-2 inhibits apoptosis and removal of auto-reactive cells, which could generate a pro-inflammatory status leading to SLE [35]. Additionally, mutations in CD95(Fas/APO-1) have been shown to predispose individuals to SLE through the loss of B lymphocyte regulation [36]. Furthermore, SLE patients display a notable infiltration of Th17 lymphocytes in their skin, which secrete various cytokines, and soluble CD95L may be involved in trafficking of Th17 lymphocytes into damaged organs [37]. Thus inhibiting the CD95/CD95L pathway could offer a promising strategy for treating diseases mediated by Th17 cells such as SLE.

#### (A.7) Vascular Endothelial Growth Factor (VEGF):

VEGF is a signaling protein that plays an important role in angiogenesis. It is produced by many cell types, such as tumor cells, macrophages, platelets, keratinocytes, and renal mesangial cells [38]. VEGF is involved in numerous processes such as lymphangiogenesis, metabolism, bone formation, hematopoiesis, and pathological angiogenesis. There have been five splicing variants of VEGF that have been identified: VEGF121, VEGF145, VEGF165, VEGF189, and VEGF206 [39]. Elevated levels of VEGF have been reported in patients with active SLE, with the highest serum levels shown in SLE patients with organ involvement such as lupus nephritis [40]. One studied show that VEGFR1 gene polymorphisms were related to risk of SLE in a Chinese Han population [41]. The VEGF −634G/C gene polymorphism was studied as well, with a result of no association of this polymorphism with SLE [42]. Due to its known involvement in SLE, VEGF serum level may be useful as a marker for disease activity and organ involvement in patients [43].

### B. Epigenetic Regulation of SLE

#### (B.1) DNA Methylation:

The most studied epigenetic change in SLE is DNA methylation, when methyl groups are added to cytosine by DNA methyltransferases [44]. DNA methylation patterns in SLE generally show global DNA hypomethylation in both T and B cells, specifically hyper-reactive CD4+ T cells, which induces an immune response and has shown to correlate with disease activity [45]. Recent studies associate specific molecular alterations such as DNA methylation with epigenetic remodeling and gene dysregulation in T cells from SLE patients, indicating a potential causative role in the disease’s pathophysiology [46]. One study identified abnormal hypermethylation in TCR co-signaling pathway genes along with higher methylation viability in BCR signaling pathways [47]. These findings could enhance our understanding of the mechanisms underlying SLE and aid in identifying new biomarkers and therapeutic targets.

#### (B.2) Histone Modifications:

Histones, which are DNA-binding proteins found in chromosomes, make up the histone code by undergoing modifications such as acetylation, phosphorylation, and methylation of histones tails [48]. Destruction of the histone code has been implicated in SLE, specifically modifications on lys9 and lys27 of H3 which can result in chromatin compacting and gene silencing. Additionally, key histone modifications are altered in immune cells, particularly CD4+ T cells and monocytes, affecting the expression of genes involved in autoimmune responses. Notably, genes like CD70 and TNF-α show increased activation markers (e.g., H3ac, H3K4me2) that correlate with disease activity, while proteins such as PP2A and HPK1 influence immune pathways by modulating IL-17 and interferon-related gene expression. Additionally, global hypoacetylation of histones H3 and H4 and dysregulation of histone-modifying enzymes are observed, contributing to abnormal immune responses and overexpression of certain immune-stimulating genes, which collectively drive SLE pathogenesis [49]. Furthermore, patients with SLE have shown reduced global histone acetylation and diminished H3K9 methylation within their CD4+ T-cells [50].

#### (B.3) Non-coding RNAs:

**Figure 4:**
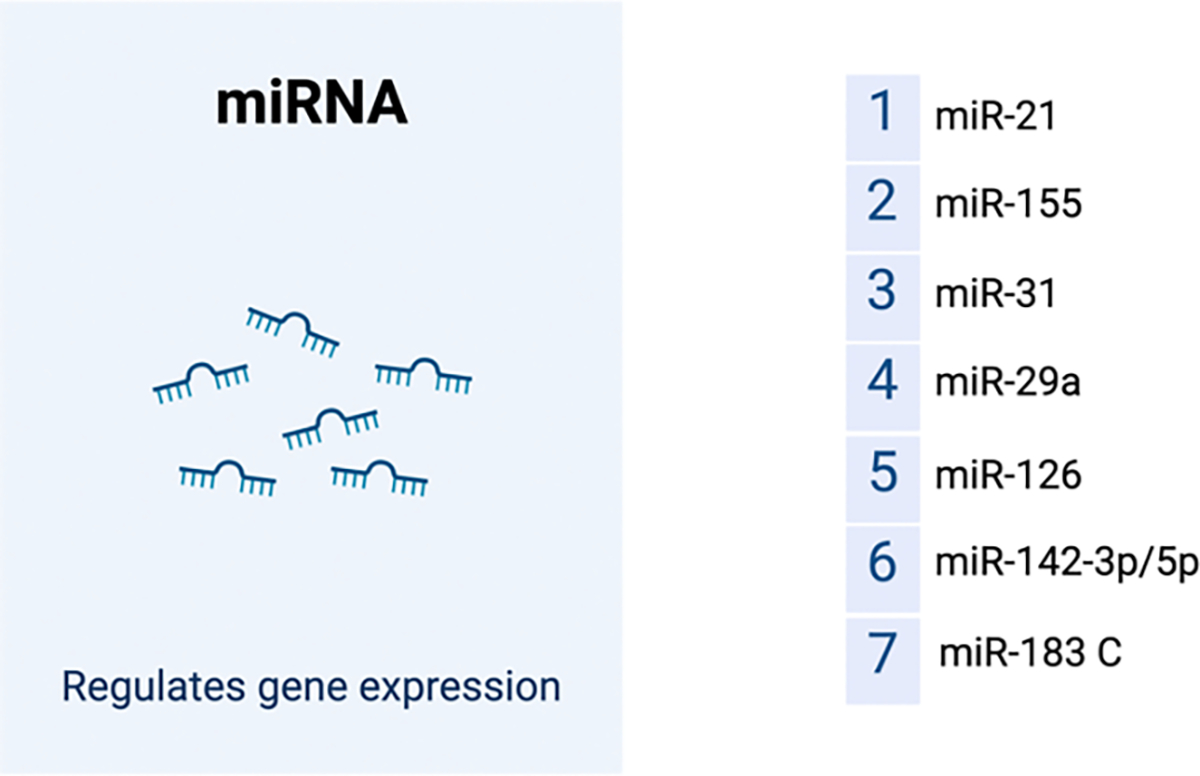
Dysregulated miRNAs implicared in SLE pathophysiology. Created with BioRender.com.

While epigenetic changes such as DNA methylation and histone modifications have been studied more extensively in relation to SLE pathophysiology, non-coding RNAs may also be implicated. The most significant subtypes of non-coding RNAs include microRNAs, long noncoding RNAs (LncRNA), and circular RNAs (circRNAs). MicroRNAs are critical in the pathogenesis of SLE, where they negatively regulate gene expression by binding to target mRNAs, thus impacting pathways like NF-κB, IFN-I, TGF, and STAT. Dysregulated miRNAs (Figure 4), as well as LncRNAs promote SLE through effects on CD4+ T cell activation and differentiation, leading to an increase in Th1, Th17, and Tfh cells and a reduction in Treg cells. Studies have also linked pregnancy to increased SLE flare-ups, likely due to miRNA-related epigenetic changes. Additionally, emerging research suggests that circRNAs contribute to SLE pathology, although more investigation into noncoding regions as risk factors is needed [51].

### C. Environmental Factors

The pathophysiology of SLE is complex and it has been postulated that environmental factors may be a factor in its complexity. One important environmental factor is UV light exposure, which can influence epigenetic modifications such as DNA methylation, which potentially exacerbates SLE. Photosensitivity, present in 73% of SLE patients, is a common diagnostic feature, with sunlight often triggering skin lesions in exposed areas. UVB light, in particular, may reduce DNMT1 expression, leading to T-cell auto-reactivity and contributing to SLE pathogenesis [52]. Epidemiological evidence links other environmental factors, such as smoking, silica exposure, and hormonal therapies, with an increased risk of developing SLE, while alcohol appears to decrease this risk. Other potential risk factors, including infections such as Epstein-Barr virus, dietary components, pollution, and certain occupational exposures, are also associated with SLE, though the mechanisms remain unclear and require further research [53].

### D. Hormonal Factors

SLE is known to be more common in men than women, especially in women of reproductive age. Additionally, male SLE patients tend to show different clinical features compared to female patients. This may be due to sex hormones, particularly estrogen and its receptor ERα, which promote autoimmune responses in SLE by enhancing autoantibody production and Th17 differentiation, and by modulating DC activation and type I IFN responses via TLR-7 and TLR-9. Androgens, such as testosterone, inhibit B cell activation but have not shown therapeutic benefits for SLE. Despite fluctuations in sex hormones during the menstrual cycle, SLE flares do not typically correlate with it. In male SLE patients, defective androgens are linked to more skin involvement and increased risk of renal damage. The use of oral contraceptives (OCPs) and hormone replacement therapy (HRT) in women with SLE carries risks, including flare-ups and cardiovascular complications, particularly for those with antiphospholipid antibodies or a history of thrombosis [54]. Therefore, hormone therapy in SLE patients should be carefully considered with close medical guidance.

### E. Nutritional Factors

There has been research on the dietary influences in SLE, which has highlighted the benefits of vitamins D and A, polyunsaturated fatty acids (PUFAs), and phytoestrogens. These have been shown to reduce proteinuria and glomerulonephritis in animal models. A balanced diet has also been shown to be important in managing SLE and addressing comorbidities like cardiovascular risk factors. Additionally, caloric restriction may benefit the immune system and reduce disease activity, while diets high in omega-3 PUFAs, such as those from fish oil or flaxseed, have anti-inflammatory effects and protect against cardiovascular complications. Moderate protein intake supports kidney function, and fiber-rich foods help regulate cholesterol and blood pressure. Vitamin D supplementation is sometimes recommended since it plays a role in immune function, especially for patients with low sun exposure. Flavonoids and isoflavones from fruits, vegetables, and soy have antioxidant and anti-inflammatory effects, while minerals like zinc, selenium, and calcium should be balanced. Curcumin, a polyphenol from turmeric, has shown potential in reducing inflammation and proteinuria in lupus nephritis. Research on the nutritional factors that may impact SLE pathophysiology offers more insight into the complex nature of the disease and the interplay of genetics and the environment [55].

### F. The Gut Microbiome

The Gut Microbiome is a new and exciting area of research in SLE. There has been recent evidence that gut microbiota dysbiosis may play some role in the pathophysiology of SLE. A study showed that patients with SLE had intestinal dysbiosis compared to healthy controls and had a lower ratio of Firmicutes/Bacteroidetes (F/B) [56]. Another study showed that the gut microbiota of those with SLE was pro-inflammatory, and that the microbiota was decreased in diversity compared to controls [57]. Additionally, research indicates that SLE patients have harmful bacteria like Ruminococcus gnavus and Enterococcus gallinarum that can disrupt the intestinal barrier. Furthermore, molecular mimicry and bacterial biofilms have been shown to be critical in SLE. Roseburia intestinalis can mimic autoantigens, stimulating autoantibody production in antiphospholipid syndrome, while biofilm-forming bacteria like Salmonella Typhimurium release DNA complexes that trigger immune cells. Since this is a relatively newer area of research, further investigations need to be made to evaluate for interventions, such as probiotics, dietary adjustments, and vaccination against specific pathogens like Enterococcus gallinarum. Other therapies such as fecal microbiota transplant, glucocorticoid treatments targeting microbiota, and mesenchymal stem cells are also being evaluated [58].

## Biologics and Targeted Therapies

Biologics have been studied for years in relation to SLE treatment. One of the reasons for this is due to the desire to move away from the use of glucocorticoids in SLE due to their side effects. There are currently two biological therapies approved for use in SLE: anifrolumab and belimumab, which are both administered intravenously. Anifrolumab is a monoclonal antibody that binds to the type-I IFN receptor (IFNAR1). It inhibits gene transcription by inhibiting the formation of an IFN/IFNAR complex [59]. Belimumab is a IgG1λ recombinant monoclonal antibody against BLys, which is a costimulator for B-cell survival and function [60]. Some studies state that anifrolumab should be used first line versus belimumab due to its faster onset of action, broad effects in SLE, and increased attainment of remission in SLE [61]. Another study showed that both anifrolumab and belimumab decreased prednisone dosages, showing a significant impact on SLE outcomes [62]. Furthermore, anifrolumab has been shown to reduce annual flare rates and time spent without flares compared to placebo [63]. Belimumab is also currently used as a treatment for lupus nephritis, whereas there needs to be more studies to evaluate the use of anifrolumab in lupus nephritis. There has been increased use of both biologics with reports from Spherix Global Insiders showing that treatment with belimumab and anifrolumab grew 32% in both the US and EU5 compared to 2022 [64]. Rituximab, a chimeric human/murine Igg1 mAb targeting CD20, is currently used off-label to treat SLE. It leads to depletion of CD20+ B cells, which has shown to be implicated in improvement in SLE disease activity [65]. Recently, the Food & Drug Administration (FDA) granted Fast Track designation to the investigational therapy AlloNK/AB-101 to be used in combination with rituximab or obinutuzumab. The Tyk2 inhibitor deucravacitinib and the antibody litifilimab, which targets plasmacytoid dendritic cells, successfully met primary endpoints in phase II trials for SLE. In contrast, ustekinumab and baricitinib achieved primary endpoints in phase II trials but did not show the same success in phase III trials [66]. This signifies exciting advances for new therapeutics in SLE.

## Future Directions

As discussed throughout this article, the pathophysiology of SLE is complex, and there are likely many factors that are involved, such as gene polymorphisms, epigenetics, environmental factors, hormonal factors, nutritional factors, and involvement of the gut microbiome. The focus of this paper was to highlight these factors and their implication in SLE to guide future research to focus on personalized medical approaches to treating patients with SLE. However, there are still gaps in our knowledge pertaining to how exactly some of these factors contribute to the pathophysiology of SLE, which has been difficult to elicit due to the complexity of SLE. Gathering more information about a patient’s gene polymorphism or epigenetics can help guide treatment options to add other medications. More research will need to be done to find effective, efficient, and cost-saving approaches. In the next 5 years, further clinical trials on biologics and therapeutics that target these gene polymorphisms and epigenetics are necessary to make a stronger claim for the best treatment option. The focus of this paper was to help guide future standard-of-care therapy to involve personalized medicine.

## Key Points:

- SLE has a significant heritable component, with specific gene polymorphisms and epigenetic modifications, such as DNA methylation and histone modifications, contributing to disease susceptibility and activity.
- SLE predominantly affects women, particularly those of reproductive age, potentially due to estrogen’s role in promoting autoimmune responses, while androgens have been shown to inhibit B cell activation.
- Factors like UV exposure, smoking, infections, and pollutants are associated with SLE onset and exacerbation, affecting immune function and epigenetic processes such as DNA methylation.
- Certain dietary elements, including vitamins D and A, omega-3 fatty acids, and phytoestrogens, may help manage SLE symptoms by reducing inflammation and supporting immune balance.
- Emerging research links gut microbiota imbalances, including harmful bacteria like Ruminococcus gnavus, to SLE pathogenesis. Gut dysbiosis, leaky gut, and bacterial biofilms may exacerbate immune responses in SLE.
- MicroRNAs and other non-coding RNAs play critical roles in SLE, regulating gene expression and affecting immune pathways like NF-κB and IFN, contributing to autoimmunity and disease progression.
- Biologic drugs like anifrolumab and belimumab are currently approved, with other therapies like rituximab, deucravacitinib, and litifilimab showing potential in clinical trials for targeting immune dysregulation in SLE.
- A personalized approach to SLE management, integrating genetics, epigenetics, and environmental factors, is necessary to enhance treatment effectiveness and minimize side effects, with more research on targeted therapies in development.
